# Radiofrequency Ablation Versus Endoscopic Submucosal Dissection in Treating Large Early Esophageal Squamous Cell Neoplasia

**DOI:** 10.1097/MD.0000000000002240

**Published:** 2015-12-11

**Authors:** Wen-Lun Wang, I-Wei Chang, Chien-Chuan Chen, Chi-Yang Chang, Lein-Ray Mo, Jaw-Town Lin, Hsiu-Po Wang, Ching-Tai Lee

**Affiliations:** From the Department of Internal Medicine (W-LW, C-YC, L-RM, J-TL, C-TL) and Department of Pathology, E-Da Hospital/I-Shou University, Kaohsiung, Taiwan (I-WC); Department of Internal Medicine, National Taiwan University Hospital, Taipei, Taiwan (C-CC, J-TL, H-PW); and School of Medicine, Fu Jen Catholic University, New Taipei City, Taiwan (J-TL).

## Abstract

Radiofrequency ablation (RFA) and endoscopic submucosal dissection (ESD) can potentially be applied for early esophageal squamous cell neoplasia (ESCN); however, no study has directly compared these 2 modalities.

We retrospectively enrolled the patients with flat-type “large” (length ≥3 cm extending ≥1/2 of the circumference of esophagus) early ESCNs treated endoscopically. The main outcome measurements were complete response at 12 months, and adverse events.

Of a total of 65 patients, 18 were treated with RFA and 47 with ESD. The procedure time of RFA was significantly shorter than that of ESD (126.6 vs 34.8 min; *P* < 0.001). The complete resection rate of ESD and complete response rate after primary RFA were 89.3% and 77.8%, respectively. Based on the histological evaluation of the post-ESD specimens showed 14 of 47 (29.8%) had histological upstaging compared with the pre-ESD biopsies, and 4 of them had lymphovascular invasion requiring chemoradiation or surgery. After additional therapy for residual lesions, 46 (97.9%) patients in the ESD group and 17 (94.4%) patients in the RFA group achieved a complete response at 12 months. Four patients (8.5%) developed major procedure-related adverse events in the ESD group, but none in the RFA group. In patients with lesions occupying more than 3/4 of the circumference, a significantly higher risk of esophageal stenosis was noted in the ESD group compared with RFA group (83% vs 27%, *P* = 0.01), which required more sessions of dilatation to resolve the symptoms (median, 13 vs 3, *P* = 0.04). There were no procedure-related mortality or neoplastic progression in either group; however, 1 patient who received ESD and 1 who received RFA developed local recurrence during a median follow-up period of 32.4 (range, 13–68) and 18.0 (range, 13–41) months, respectively.

RFA and ESD are equally effective in the short-term treatment of early flat large ESCNs; however, more adverse events occur with ESD, especially in lesions extending more than 3/4 of the circumference. RFA does not allow for pathology to evaluate the curability after ablation, and thus currently the use for invasive ESCNs should be conservative until longer follow-up studies are available.

## INTRODUCTION

Esophageal cancer is a common and highly lethal disease, causing more than 400,000 deaths per-year worldwide.^1^ In the Asia-Pacific region, squamous cell carcinoma is the major histological type of the disease, and its incidence continues to rise in Taiwan.^1,2^ Recent advances in image-enhanced endoscopy have enabled an early accurate diagnosis of esophageal squamous cell neoplasia (ESCN).^3,4^ For the treatment of early ESCNs, endoscopic submucosal dissection (ESD) enables en bloc resection of the neoplasia, and the resected specimen allows for a pathological assessment to evaluate the curability.^5–8^ However, ESD is a complicated procedure that requires a high level of expertise, especially for large lesions.^7,8^ In addition, a long learning period is required to successfully perform ESD, and therefore this procedure can only be performed in high capacity institutes. In addition, esophageal strictures have been reported to complicate more than 90% of cases of esophageal ESD involving the entire lumen circumference.^8–10^ The resultant dysphagia substantially decreases the patients’ quality of life, requiring multiple sessions of endoscopic dilatation.^11^ On the other hand, radiofrequency ablation (RFA) is a more convenient and safe method, and recent studies have shown its efficacy and safety in treating dysplasia in cases of Barrett's esophagus.^12–15^ In addition, several studies including our pilot series have recently demonstrated its potential to treat early ESCN.^16–20^ However, whether RFA can be an alternative to ESD for the treatment of ESCN is still uncertain. Therefore, this study aimed to compare the efficacy and safety of RFA and ESD in treating large early ESCNs.

## MATERIALS AND METHODS

### Patients and Design

We performed a retrospective cohort study to enroll patients from July 2009 to December 2013 that met the following inclusion criteria. Results of Lugol's chromoendoscopy showed unstained or mosaic-like lesions that occupied more than 50% of the circumference of the esophagus and extended more than 3 cm longitudinally. Histological results revealed squamous high-grade dysplasia or mucosal squamous cell carcinoma. The esophageal lesion was completely flat (type 0-IIb), according to the Paris classification of the endoscopic appearance of early gastrointestinal neoplasias.^21^ Endoscopic ultrasound showed no submucosal invasion or lymphadenopathy. Computed tomography revealed no metastasis or lymphadenopathy. The patient provided informed consent.

Patients were excluded if any of the following exclusion criteria were met. Having a stricture that prevented the passage of a therapeutic endoscope. A history of endoscopic resection, surgery or radiation of the esophagus. Uncontrolled coagulopathy with an international normalized ratio >2 or platelet count <75,000 per μL. Decompensated cirrhosis (Child-Pugh score ≥ 7). Having large varices or small varices with red wale marks, or a history of variceal bleeding. Magnifying endoscopy showed the intra-epithelial papillary capillary loop as type V3 (m3 invasion) or Vn (submucosal invasion) pattern, according to the classification of microvascular architecture of superficial esophageal carcinoma.^22^ The subject was pregnant. The Institutional Review Board of E-Da Hospital approved the study.

**FIGURE 1:**
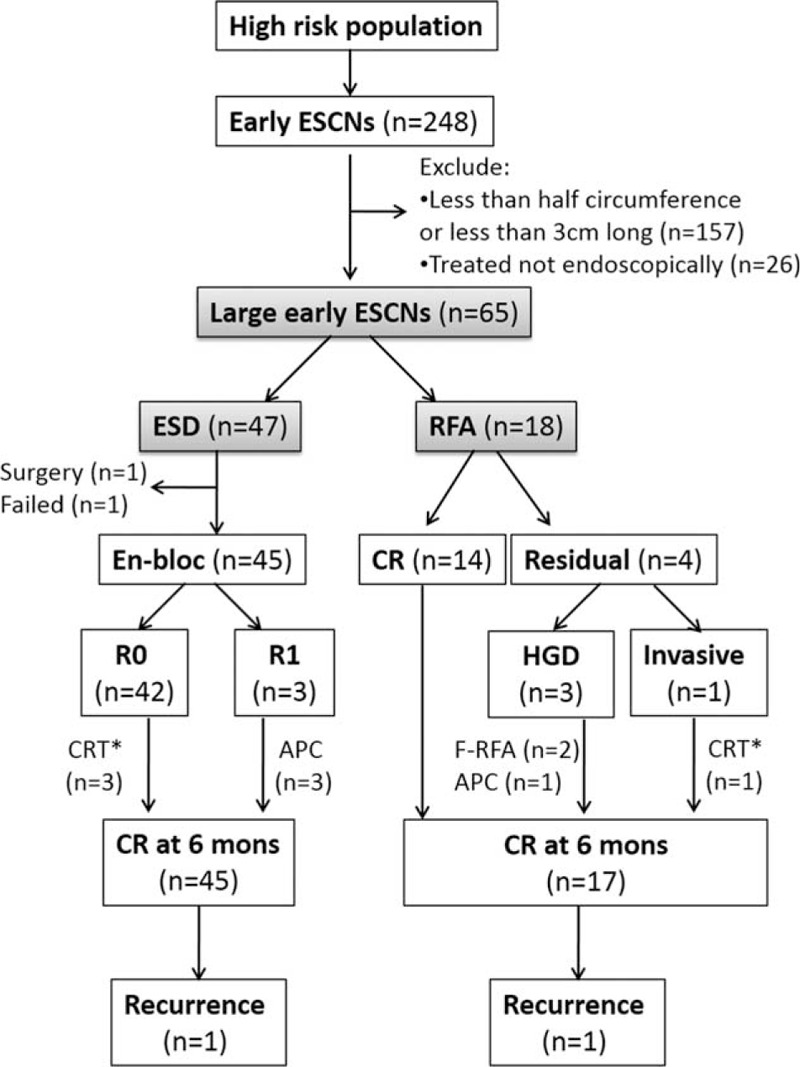
Flowchart of patient enrollment for analysis. ^∗^Additional chemoradiation therapy for poor histological features, such as lymphovascular invasion or postablation histology revealed invasive cancer to the muscularis mucosa layer. APC = Argon plasma coagulation, CR = complete remission, ESCNs = esophageal squamous cell neoplasias, F-RFA = focal-type (HALO90) radiofrequency ablation, HGD = high grade dysplasia.

The flowchart of patient enrollment is shown in Figure 1. Before July 2011, all of the patients received ESD as the initial treatment, and RFA was available at our institute from July 2011 onward. The patients could choose which treatment modality they preferred. All patients provided informed consent following a full explanation of the use of RFA or ESD and alternative treatment options. A complete medical history was obtained before the endoscopic procedures, which included demographic and clinical data. Alcohol drinkers, betel nut chewers, and cigarette smokers were defined as those consuming any alcoholic beverage during a week, those who chewed more than 7 betel nuts per week, and those who smoked more than 10 cigarettes per week for at least 6 months, respectively.^23^

### Endoscopic Procedure With ESD or Circumferential Balloon-Based RFA

The endoscopic procedures were performed using conscious sedation or anesthesia with appropriate doses of midazolam, fentanyl, and/or propofol. Prior to performing ESD or RFA, Lugol's staining was performed to determine the location and size of the Lugol-voiding lesions. The area from 1 cm proximal to 1 cm distal to the Lugol-voiding lesion-bearing segment of the esophagus was defined as the treatment area, and was marked by taking 2 biopsies or argon plasma coagulation (APC).

**FIGURE 2:**
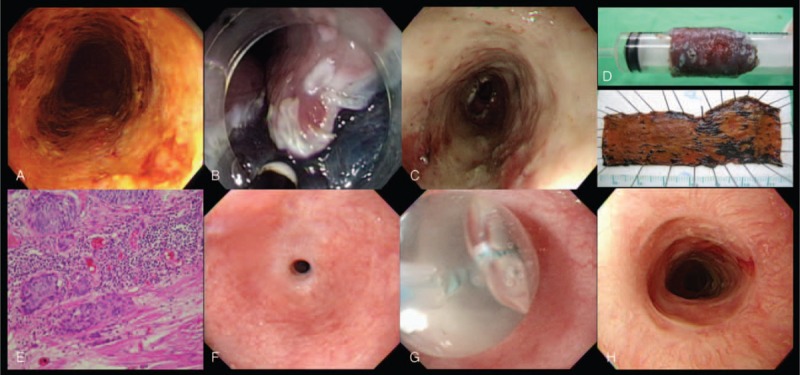
Endoscopic submucosal dissection of early esophageal squamous cell neoplasia. (A) Detection of the near total circumferential lesion (unstained part) using Lugol's chromoendoscopy; (B) ESD was performed using an IT-2 knife. (C) artificial ulcer after the removal of the lesion; (D) resected en bloc specimen; (E) histological evaluation of the resected specimen showed squamous cell carcinoma with invasion to muscularis mucosa; (F) severe stricture developed after ESD; (G) balloon dilatation for the stricture; and (H) Esophageal stricture resolved after multiple sessions of dilatation. ESD = endoscopic submucosal dissection.

The ESD procedure (Fig. 2) was performed by a single endoscopist (C-TL) as described in our previously report.^7^ Briefly, a distal attachment (D-201-11802; Olympus Co. Ltd., Tokyo, Japan) was attached to the tip of the endoscope to obtain a constant endoscopic view and to create tension on the connective tissue for the submucosal dissection. A 23-gauge disposable injector was then used to inject 3 to 5 mL glycerol solution plus indigo-carmine with 0.0025% epinephrine into the submucosa to lift the lesion. A circumferential incision was made initially, followed by a submucosal dissection with an IT-knife 2 (KD-610L; Olympus Co. Ltd.). To control bleeding, hemostatic forceps (FD-410LR; Olympus Co. Ltd.) were used in a soft coagulation mode (60 W output). After endoscopic dissection, the resected specimens were evaluated. In the patients with poor histological features (ie, invasion in m3 or deeper or lymphovascular invasion), adjuvant surgery or chemoradiation therapy was suggested.

**FIGURE 3:**
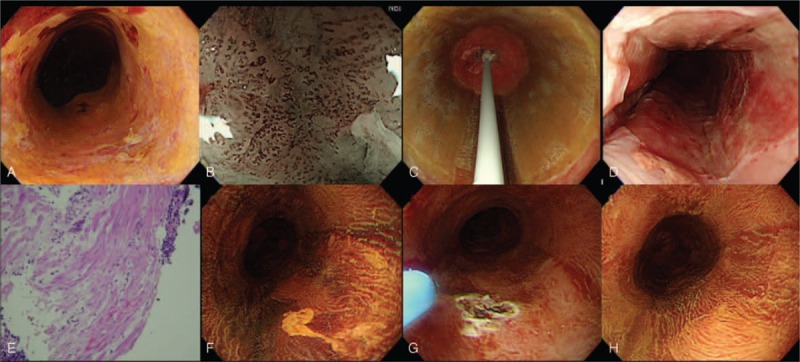
Circumferential balloon-based radiofrequency ablation of early squamous neoplasia. (A) Lugol's staining showed a circumferential unstained lesion; (B) pretreatment evaluation with narrow band imaging and magnifying endoscopy to demonstrate the pattern of intra-epithelial papillary capillary loop (IPCL); (C) circumferential ablation catheter placed in the esophagus to ablate the lesion; (D) appearance of the mucosa after the second ablation; (E) the histology of endoscopic biopsy taken over the treatment area immediately after the RFA procedure, demonstrated the muscularis mucosa layer without viable tumor. (F) At 3 months, a small residual Lugol unstained lesion was noted and further eradicated with argon plasma coagulation (G); (H) 6 months after primary circumferential RFA, Lugol's staining showed no evidence of residual squamous neoplasia. A biopsy also confirmed the complete response. RFA = radiofrequency ablation.

All primary RFA (Fig. 3) were performed by a single endoscopist (W-LW), using a HALO360 System (Covidien GI Solutions, Sunnyvale, CA), which has been approved by the US Food and Drug Administration (FDA) and is approved for use in Europe (CE mark) and Taiwan (Ministry of Health and Welfare). The HALO360 system consists of an ablation catheter, an energy generator, and a sizing balloon. The ablation catheter balloon is encircled by a 3-cm-long bipolar array that delivers short-duration RFA at 40 W/cm^2^ and 10 to 12 J/cm^2^. We used an ablation (12 J/cm^2^)-clean-ablation (12 J/cm^2^) regimen for all of the procedures (Fig. 3).^17,20^ Immediately after the RFA procedure, at least 4 endoscopic biopsies every 2 cm were taken over the treatment area. If the post-treatment histology revealed invasive cancer to the muscularis mucosa layer, a rescue therapy (surgery or chemoradiation) was suggested.

### Outcome Measures and Follow-Up

After the endoscopic procedures, the patients were administered esomeprazole 40 mg daily, and sucralfate suspension 5 mL (200 mg/mL) 4 times daily for 1 month. Acetaminophen and an oral narcotic were prescribed as needed. The patients then received follow-up endoscopy with image-enhancing modalities including Lugol's staining and narrow-band imaging. This was performed 1, 3, and 6 months after the procedure and every 6 months thereafter. Two biopsies were taken endoscopically from the normal appearing mucosa over the treatment area and from Lugol's unstained areas or any suspicious lesions. If residual squamous intraepithelial neoplasms were detected during follow-up endoscopy, focal ablation with the HALO90 System (12 J/cm^2^, 2 applications) or APC (Olympus ENDOPLASMA, 1.5 L/min, 35 W) was performed for residual high-grade dysplasia/mucosal cancer.

The primary endpoint was the proportion of patients with a histologically complete response at 12 months, defined as the absence of squamous neoplasia from any biopsy specimen taken from the treatment area. There were 3 secondary endpoints.The first is the degree of adverse events, including massive bleeding, emphysema, and perforation during the procedure. Massive bleeding was defined as blood loss >500 mL or a hemoglobin drop >2 g/dL. Perforation was diagnosed when other organs, extraluminal fat, or the extraluminal space were observed endoscopically through the muscle layer during the procedure. Mediastinal emphysema was diagnosed by the presence of air in the mediastinal space. A postoperative stricture was defined if a standard 9.8-mm diameter endoscope failed to pass through the stenosis. Second, the procedure time of both modalities. The procedure time of ESD was the time required for both the circumferential incision and submucosal dissection, and that of RFA as the time from insertion of the sizing balloon to completion of ablation. Third, the proportion of patients demonstrating neoplastic progression during follow-up.

### Statistical Analysis

All statistical analyses were performed using SPSS software (SPSS for Windows, version 18.0; SPSS Inc., Chicago, IL). Comparisons between the different treatment groups and the clinical characteristics were performed using the χ^2^ test or *t* test or Wilcoxon Rank-Sum nonparametric test as appropriate. A *P* value < 0.05 was considered to indicate statistical significance.

## RESULTS

### Patients and Endoscopic Characteristics

A total of 65 patients with large, early flat ESCNs were consecutively enrolled (Fig. 1), 18 of whom received circumferential balloon-based RFA and 47 received ESD. The demographics and tumor characteristics of these patients are shown in Table 1. There were no significant differences in average age, gender distribution, habits of substance use, mean body mass index, and the pretreatment evaluation of tumor invasion depth between the 2 groups. The patients treated with RFA tended to have a larger tumor size (72.2 vs 57.3 mm; *P* = 0.13) and larger circumferential extension of the tumor than those treated with ESD (*P* = 0.01).

**TABLE 1:**
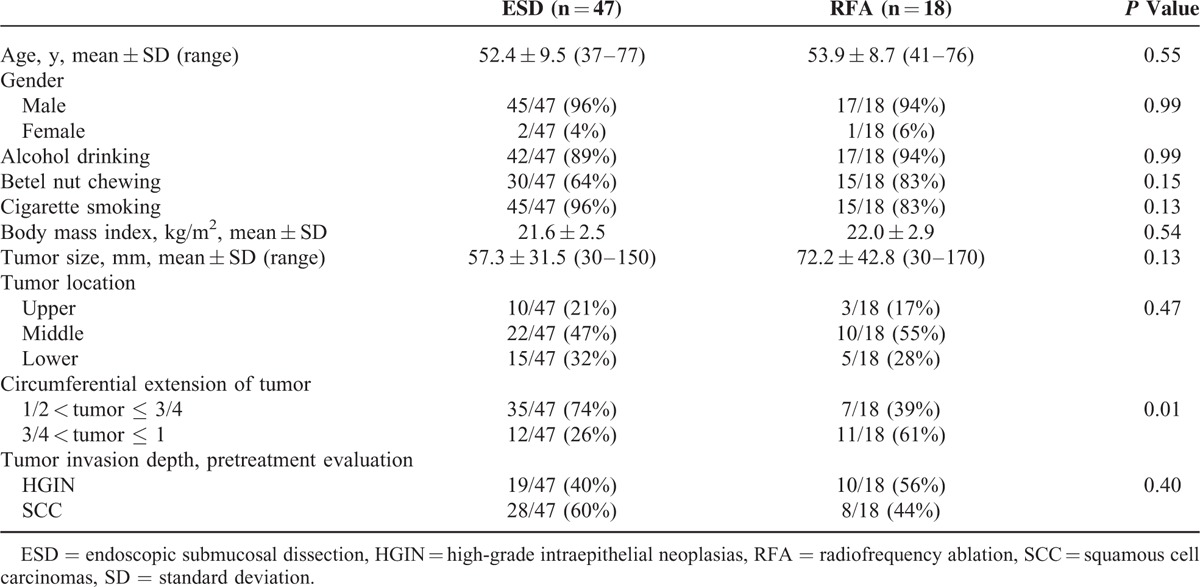
Demographic and Endoscopic Characteristics

### Efficacy of Endoscopic Treatment

As shown in Table 2, the mean procedure time of ESD was significantly longer than that of RFA (126.6 vs 34.8 min; *P* < 0.001), even though the treatment area was larger in the RFA group (64.4 vs 89.4 mm; *P* = 0.011). Among those who received ESD, 45 (95.7%) achieved en bloc resection and 42 (89.3%) had complete resection (R0 resection). Of the patients with incomplete ESD, 1 received an emergency operation due to an uncontrolled perforation, and 1 refused further treatment. Three received APC for residual lesions. The histological evaluation of the resected specimens showed that 14 of 47 (29.8%) had histological upstaging compared with the pre-ESD biopsies (Table 3). Twelve of 47 (25.6%) had poor histological features (12 had m3/sm1 invasion, 4 of which had lymphovascular invasion).

**TABLE 2:**
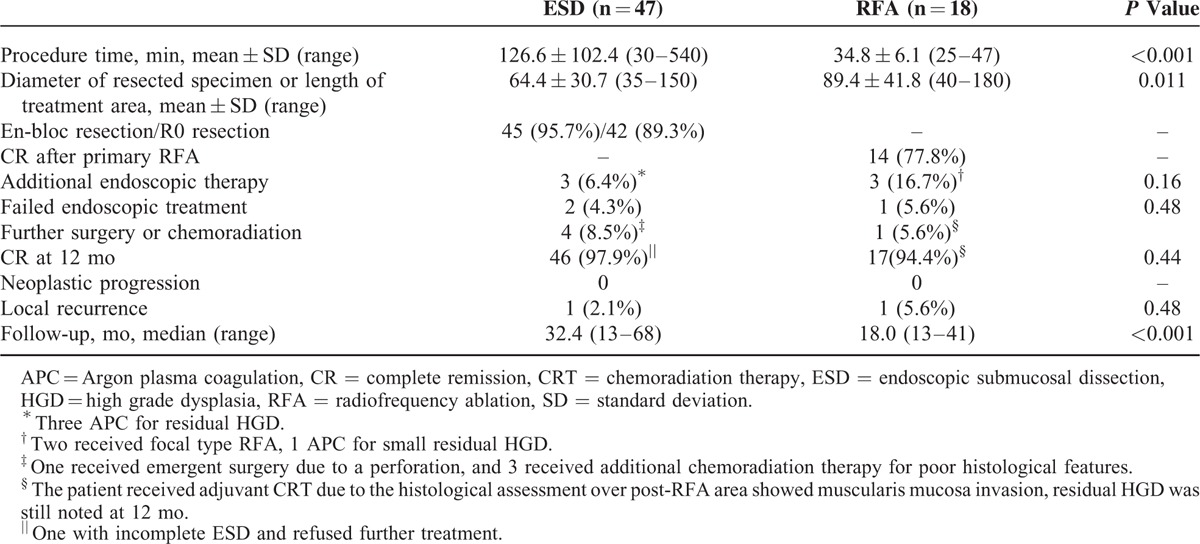
Efficacy of ESD and RFA Procedures

**TABLE 3:**
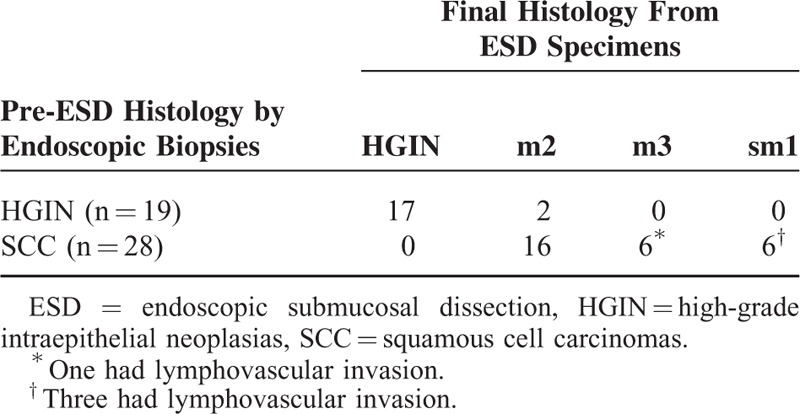
Comparison of the Histology From Pre-ESD Endoscopic Biopsies and the ESD Resected Specimens

Among the patients with lymphovascular invasion, 3 received additional adjuvant chemoradiation therapy and 1 underwent surgery (the patient who underwent an emergency operation). At 12 months, 46 of the 47 (97.9%) patients had achieved a complete response, with intention-to-treat analysis.

Of the 18 subjects who received RFA, 14 (77.8%) achieved a complete response after primary RFA. Three patients had residual high-grade dysplasia at 1 month which was eradicated with focal-type RFA (n = 2) or APC (n = 1). One patient was found to have invasive cancer of the muscularis mucosa in a histological evaluation immediately postablation, and rescue therapy with chemoradiation was performed. After these additional therapies, 17 of the 18 (94.4%) patients achieved a complete response at 12 months. One patient who received rescue chemoradiation therapy had residual high-grade dysplasia. There was no neoplastic progression in either group during the study period; however, 1 patient who received ESD and 1 who received RFA developed local neoplastic recurrence over the treatment area during a median follow-up period of 32.4 (range, 13–68) and 18.0 (range, 13–41) months, respectively.

### Adverse Events of the Endoscopic Treatment

In the ESD group, 4 patients developed major adverse events (8.5%), including 1 with mediastinal emphysema, 1 with bleeding, and 2 with perforations. Sixteen patients (34.0%) had stenosis after ESD that required balloon dilatation with a median of 8 sessions (range, 2–65; Table 4). In contrast, there were no procedure-related major adverse events in the RFA group, although 4 (22.2%) developed stenosis that needed a median of 5.5 sessions of dilatation (range, 2–8). There were no cases of procedure-related mortality in either group. Notably, in the patients with lesions that occupied more than 3 quarters of the circumference, a significantly higher risk of stenosis was noted in the ESD group compared with the RFA group (83% vs 27%, *P* = 0.01). Post-ESD stricture also required more sessions of balloon dilatation to resolve the dysphagic symptoms than post-RFA stenosis (median, 13 vs 3; *P* = 0.04).

**TABLE 4:**
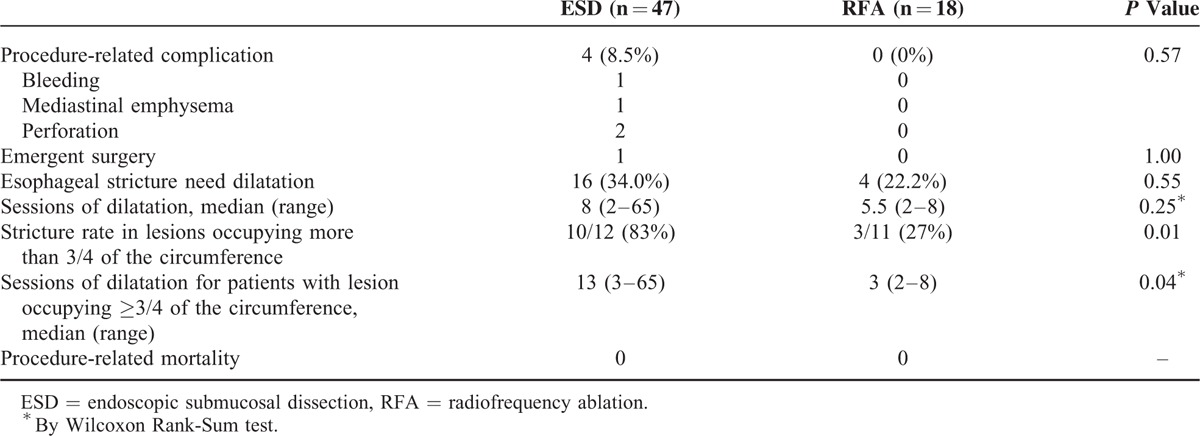
Safety of ESD and RFA Procedures

## DISCUSSION

ESD of large early ESCN requires considerable expertise, and may lead to esophageal strictures. Several recent studies have demonstrated the potential of RFA to treat early ESCN; however, whether RFA can be an alternate to ESD is still uncertain. The present retrospective cohort study demonstrated that both RFA and ESD had good efficacy to treat large, flat, early ESCNs. For lesions extending more than 3 quarters of the circumference, RFA is superior to ESD with regard to a shorter procedure time and lower stricture rate following removal of larger lesions. To the best of our knowledge, this is the first study to directly compare the efficacy and safety of ESD and RFA for the treatment of ESCNs.

While ESD is gaining popularity, it remains a time-consuming technique that is associated with higher rates of adverse outcomes, including massive bleeding, perforation, and postprocedural strictures. The size and depth of invasion of the lesion and the excision extension of the lumen strongly relate to incidences of adverse events.^9^ In our study, the absence of delayed bleeding and lower rate of adverse events may be related to the high level of expertise of the endoscopists. To be able to perform ESD well and safely involves a significant learning curve,^24^ which has limited the use of this technique worldwide and explains why it is only used in select high-volume centers. However, RFA appears to be less technically demanding, with a minimal learning curve^25^ compared with ESD, and thus may be more feasible for less experienced endoscopists. RFA is already the first treatment of choice for dysplasia in cases of Barrett's esophagus in many countries.^14,15^

Esophageal stenosis is still a serious concern after ESD, especially for those with lesions involving more than 3 quarters of the circumference. Stenosis may decrease the quality of life and require multiple dilatations to resolve the symptoms, and multiple dilations may increase the frequency of adverse events including esophageal perforation. The present study also demonstrated a high rate of stenosis after ESD (83%), which is compatible with previous reports.^10,26^ We found a lower stenosis rate (27%) for the patients treated with RFA, and these patients also required fewer sessions of dilatation (median, 3 vs 13; *P* = 0.04). We previously demonstrated the good efficacy of RFA for the treatment of ultra-long, extensive early ESCNs.^20^ Therefore, we believe that RFA has the potential to be a convenient technique for treating large early ESCNs. It does not require a high level of endoscopic expertise, and is associated with a low rate of adverse event. Furthermore, the risk of stenosis was higher in our cohort than that reported after RFA for Barrett's esophagus (0–9%) or ESCNs (14%).^13,18,27^ A recent study reported that the rate and severity of stenosis after RFA in porcine squamous esophagi increased when preceded by Lugol staining.^28^ Routine Lugol staining before RFA may partly explained the relatively higher rate of stenosis in our cohort. In addition, we also analyzed the predictors for post-RFA stenosis, and found a longer ablation length was associated with a higher risk of stenosis. Similarly, for Barrett's esophagus, a previous study also reported that a long segment length may increase the risk of strictures.^27^

When making decisions regarding the choice of endoscopic treatment, clinicians must consider the merits of each technique. Complete ESD has the advantage of providing whole resected specimens for histological evaluation. In our cohort with large flat early ESCNs, all of the subjects with noninvasive neoplasias in pretreatment histological evaluation achieved a complete response at 12 months in both groups. Due to the tissue-destructive modality of treatment, RFA does not allow for pathology to evaluate the curability after ablation. This is a major concern, and thus the use for SCC should be conservative. Currently, the optimal depth of ablation when using RFA for squamous neoplasia is still unclear. Only 1 previous study has been published on this issue, which reported that the ablation depth in humans over the normal squamous epithelium in 3 of 5 patients achieved histological ablation to the muscularis mucosa (m3) layer with 2 ablations of 12 J/cm^2^.^29^ Even though a recent UK study demonstrated that 30% of patients with early squamous cell neoplasias progressed to invasive cancer after only 1 ablation treatment.^19^ However, in our study using a double ablation regimen, no patient exhibited neoplastic progression during a mean follow-up period of 13.1 months. Therefore, treatment using RFA with a 12 J/cm^2^—clean—12 J/cm^2^ regimen may be effective and indicated for HGIN (high-grade intraepithelial neoplasias) or m2 (lamina propria invasion) esophageal squamous cell carcinomas (ESCCs). However, in our cohort, we found 14 of 47 (29.8%) had histological upstaging in the final resected specimens compared with the pre-ESD biopsies. The significant upstaging would highlight the limitations of relying on endoscopic biopsies to appropriately stage patients and decide who may be an RFA candidate. Despite meticulous pretreatment evaluations using conventional endoscopy, endoscopic ultrasound, computed tomography, and magnifying endoscopy,^4,30^ we found that 12 of the 47 (25.6%) patients who received ESD had poor histological features in the resected specimens. Four of them had lymphovascular invasion and additional treatment was suggested. This finding indicates that a relatively high percentage of patients needed rescue therapy, and thus RFA should be used conservatively for invasive ESCCs. Although we defined a protocol using a histological evaluation immediately postablation to serve as an indicator for the need of additional treatment and almost all patients achieved a complete response at 12 months, long-term follow-up is needed to confirm the outcomes of RFA for the treatment of early ESCCs. The risk of incomplete ablation and recurrence of ESCNs after treatment reiterates the need for strict patient selection and continuing endoscopic surveillance. Further research with a longer follow-up period is needed to determine the optimal rescue protocol and long-term outcomes after RFA. Given the high rate of poor histological features on the ESD specimens, we currently suggest RFA may be option for high-grade intra-epithelial neoplasia, but more data are needed before RFA can be considered as a primary treatment option for m2 ESCCs.

There are several limitations to this study. First, the study is inevitably limited by its retrospective nature. Nevertheless, based on our results, a future randomized study to compare RFA with ESD is justified, especially for those with large noninvasive neoplasias. Second, the sample size is small, and the study was conducted at a single institution. Third, because RFA only became available in our institute in July 2011, the follow-up period is limited. A longer follow-up study is needed to verify the efficacy of these treatment modalities.

In conclusion, this study demonstrated that RFA and ESD are equally effective in the short-term treatment of early flat large ESCNs; however, ESD was associated with a higher rate of adverse events, especially in the lesions extending for more than 3 quarters of the circumference. Based on these results, a randomized study to compare RFA with ESD is justified, especially for the patients with large noninvasive squamous neoplasias.
